# A rolling stone: vomiting of a gallstone without the presence of a biliary-enteric fistula

**DOI:** 10.1093/omcr/omaa125

**Published:** 2021-01-23

**Authors:** Athina A Samara, Konstantinos Perivoliotis, Ioanna-Konstantina Sgantzou, Alexandros Diamantis, Theodoros Floros, Dimitrios Symeonidis, Konstantinos Tepetes

**Affiliations:** 1 Surgery Department, University Hospital of Larissa, Thessaly, Greece; 2 Radiology Department, University Hospital of Larissa, Thessaly, Greece

**Keywords:** gallstone vomiting, gallstone disease, cholelithiasis

## Abstract

Gallstones may pass into the gastrointestinal tract spontaneously through the ampulla of Vater or through a biliary-enteric fistula. This report describes an extremely rare case of a patient vomiting a gallstone without the presence of a fistula between the gallbladder and the gastrointestinal tract. Furthermore, no imaging findings of gallstones disease appeared. The patient has been treated conservatively and all symptoms subsided. The patient remains asymptomatic 3 months after treatment and an elective laparoscopic cholecystectomy was arranged. Including this reported case, only three cases have been described in the literature worldwide. However, our case is the only one characterized by retrograde flow of the gallstones into the stomach without symptoms of bowel obstruction or other underlying pathologies.

## INTRODUCTION

Gallstone disease is among the most common intra-abdominal conditions resulting to hospital admissions in developed countries. More specifically, gallstone disease affects up to 15% of the population and 10–15% of them will, ultimately, develop choledocholithiasis [1,2].

In cases of symptomatic gallstone disease, among the mechanisms involved, gallstone migrates through the bile duct, causing obstruction [3,4]. It is estimated that ~5 in 1000 people will experience biliary obstruction symptoms and jaundice. Pain located in the epigastrium and right hypochondrium is associated with the presence of gallstones. However, functional symptoms, such as acid regurgitation, nausea and digestive disorders that were considered as typical symptoms of gallstone disease, in recent cohorts, were not associated with the presence of gallstones [4].

Besides choledocholithiasis, inflammatory or malignant lesions involving segments of the biliary tree can be presented with the symptoms of biliary obstruction [5]. However, differential diagnosis can be sometimes difficult due to the overlap of clinical and laboratory findings, alongside the non-conclusive reports of the applied imaging modalities [5].

This report describes the extremely rare case of a patient presenting with biliary obstruction, attributed to an intraductal tumor that ameliorated after vomiting a gallstone.

## CASE REPORT

A 63-year-old Caucasian male presented at the emergency department of a secondary hospital with fever (up to 38°C) and abdominal pain. Symptoms had developed over the previous 6 days. The patient’s medical history included hypertension, hypothyroidism, coronary disease and hyperlipidemia. Physical examination did not identify any pathological finding. Routine laboratory examinations confirmed increased inflammatory markers (white blood cells: 10 300/μl, C-Reacting Protein: 22 mg/l) and a biliary obstruction pattern (total bilirubin: 1 mg/l, alkaline phosphatase: 123 IU/l, γ-GT: 456 IU/l, amylase: 178 IU/l and serum glutamic oxaloacetic transaminase/serum glutamic pyruvic transaminase: 51/110 IU/l). Ultrasonography revealed dilation of the common bile duct, without, though, any stones within the gallbladder. The patient was admitted to the hospital for further investigation.

**Figure 1:**
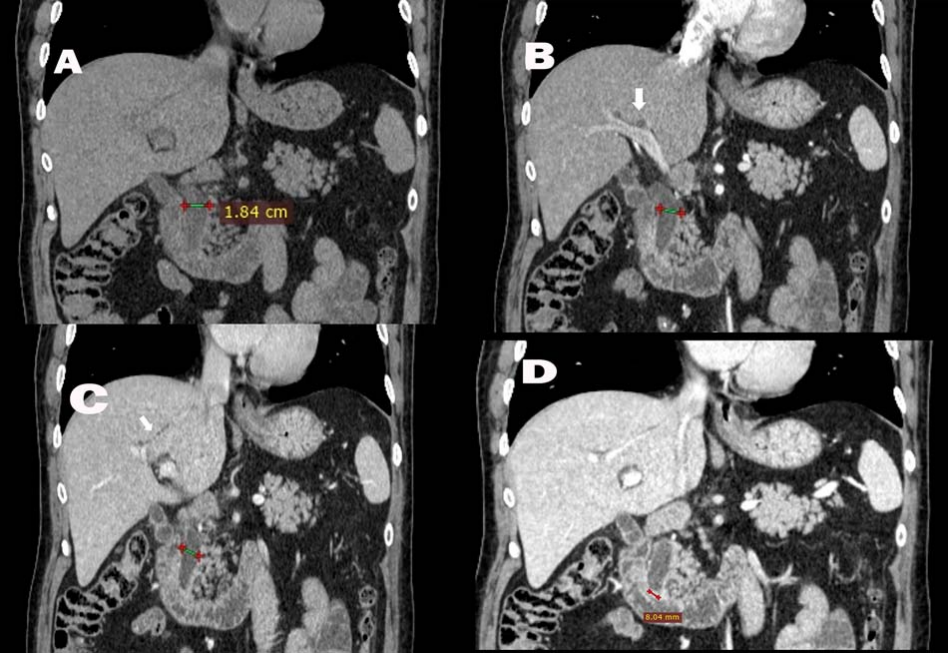
Quadruple-phase computed tomography. (A) Pre-contrast, (B) arterial phase and (C) venous phase Coronal CT (soft tissue window) have shown an intrahepatic (white arrow) and extrahepatic dilation of the biliary tree, with common bile duct (crosses) measuring 18.4 mm. No image of intrabiliary hyperdense/hypodense or contrast enhanced tissue was shown. (D) In the delayed phase of Coronal CT (soft tissue window), an enhanced tissue in the common bile duct, with a maximum diameter of 9 mm, was identified.

A pancreatic protocol computer tomography scan was conducted the next day and revealed both intrahepatic and extrahepatic dilations. The gallbladder was normal without lithiasis, and the common bile duct measured at 18.4 mm. Before the ampulla of Vater, a low attenuation mass with a marked enhancement with a maximum diameter of 9 mm was identified (Fig. 1). In addition, all tumor markers were within normal ranges. These findings were compatible with an intraductal lesion and the patient was referred to our tertiary center for further management.

**Figure 2:**
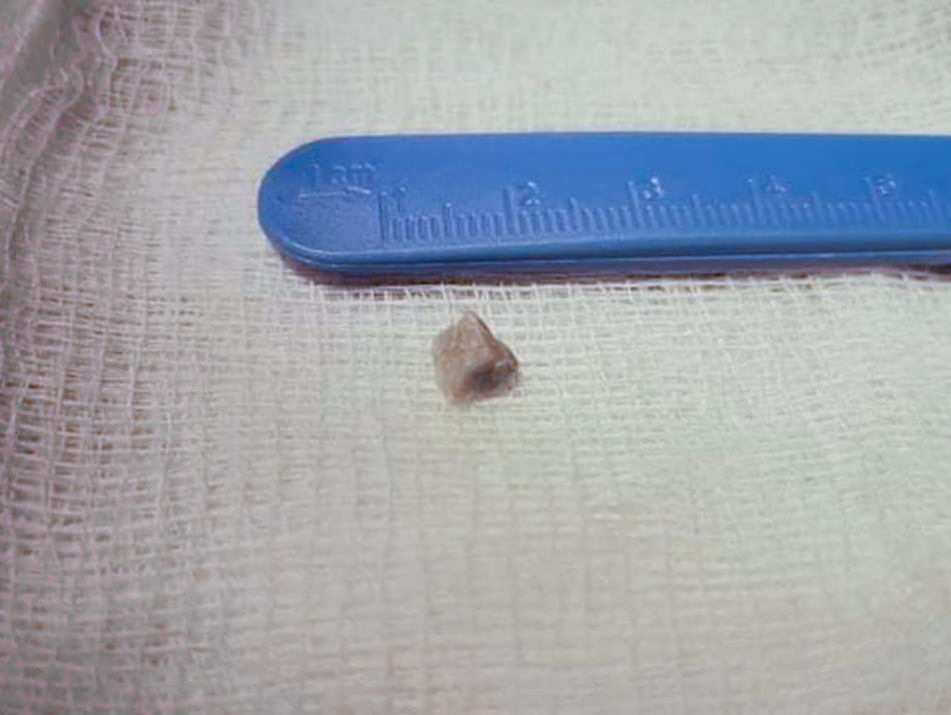
The gallstone inside the vomitus.

**Figure 3:**
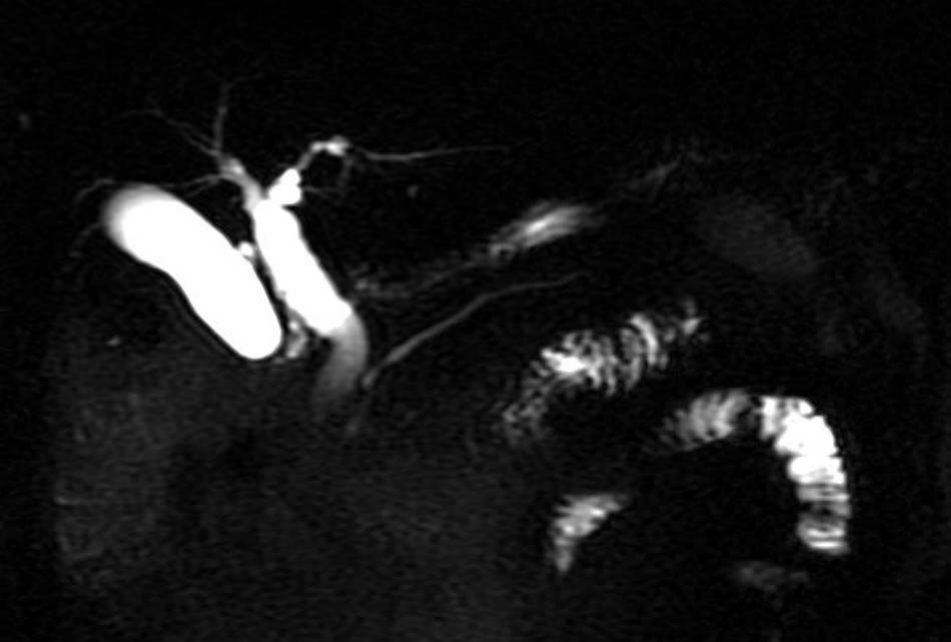
Magnetic resonance cholangiopancreatography: no filling defects within the intrahepatic or extrahepatic biliary tree. The common bile duct measures 12 mm, without the presence of extra tissue.

During the first day of admission, the patient experienced an acute episode of epigastric pain; half an hour later, he had a bilious vomiting episode with a gallstone within the vomitus (Fig. 2). Following this episode, all symptoms dissipated and the patient’s laboratory examinations returned to normal ranges. A magnetic resonance cholangiopancreatography was conducted in order to re-assess the previous findings and it revealed no filling defects. The common bile duct measured at 12 mm (Fig. 3). A subsequent upper gastrointestinal (GI) endoscopy reported a dilated ampulla, a finding indicative of a recent gallstone passing. Moreover, no signs of an underlying fistula were found. Two days later, the patient was discharged without any symptoms.

During follow-up, an endoscopic ultrasonography was performed, with no pathological findings from the pancreas, the common bile duct or the ampulla of Vater. The patient remained asymptomatic 3 months later and an elective laparoscopic cholecystectomy was arranged.

## DISCUSSION

Gallstones may pass into the GI tract spontaneously through the common bile duct, particularly if the stone size is <3.5 mm [6]. However, a stone can also pass through a biliary-enteric fistula, a complication seen in 0.3–1.5% of all patients with cholelithiasis [7]. In such a case, stones can obstruct the bowel lumen, thus resulting to a gallstone ileus. In our case, a 9-mm stone obstructed the final part of the biliary tract and passed through, without the presence of a fistula or a previous sphincterotomy. Moreover, another interesting characteristic was that initial imaging diagnosis was consistent with an intraductal mass.

CT pancreatography is now the gold standard protocol for pancreatico-biliary malignancies, with a sensitivity of 72–78% for the detection of biliary stones [5, 8]. Similarly to our case, biliary stones may be visible in a CT as hyperattenuating lesions surrounded by hypoattenuating bile and ampullary soft tissue. Moreover, distal choledocholithiasis may cause papillitis, with radiographic findings of bile duct obstruction at the papilla demonstrated by smooth and symmetric papillary edema, as presence of soft tissue, and contrast enhancement [5]. The differential diagnoses between benign and malignant conditions in the ampulla and periampullary region are difficult to assess radiologically as many imaging features are overlapping. Papillary size of less than 12.3 mm was identified as the only independent variable differentiating benign from malignant causes of papillary stenosis [5].

The vomiting of gallstones without the presence of a biliary-enteric fistula is extremely rare. To date, only two other cases have been reported in the literature. The first case occurred in 1951 and involved a 65-year-old female vomiting a 0.5-cm facetted gallstone [9]. The second case was reported in 2013 and involved an 83-year-old female. The patient experienced multiple episodes of vomiting gallstones, secondary to an inflammatory stricture causing obstruction without the presence of a cholecysto-duodenal fistula [10].

To the best of our knowledge, including the present patient, only three cases of vomiting a gallstone, without the presence of a biliary-enteric fistula, have been reported in the literature. Besides these, the complexity of our case was further enhanced by the fact that preliminary reports were consistent with an intraductal neoplasia, thus complicating initial diagnostic approach. Moreover, no other risk factor for a retrograde flow of the gallstones into the stomach was identified. Despite the remarkable size of the stone, the pathology was self-resolved without any complication, thus avoiding an emergency operative management.

Herein, we presented the extremely rare case of a patient presenting with biliary obstruction due to a gallstone mimicking an intraductal tumor. Despite the lack of a biliaro-enteric fistula, the stone passed and was vomited, resulting to the amelioration of symptoms.

## References

[1] Tazuma S Gallstone disease: epidemiology, pathogenesis, and classification of biliary stones (common bile duct and intrahepatic). Best Pract Res Clin Gastroenterol 2006;20:1075–83. 17127189 10.1016/j.bpg.2006.05.009

[2] Aerts R, Penninckx F The burden of gallstone disease in Europe. Aliment Pharmacol Ther 2003;18:49–53. doi: 10.1046/j.0953-0673.2003.01721.x14531741. 14531741

[3] Nassar Y, Richter S Management of complicated gallstones in the elderly: comparing surgical and non-surgical treatment options. Gastroenterol Rep 2019;7:205–11. 10.1093/gastro/goy046. PMC6573799 31217985

[4] Shabanzadeh DM Incidence of gallstone disease and complications. Curr Opin Gastroenterol 2018;34:81–9. doi: 10.1097/MOG.0000000000000418 PMID: 29256915. 29256915

[5] Nikolaidis P, Hammond NA, Day K, Yaghmai V, Wood CG 3rd, Mosbach DS et al. Imaging features of benign and malignant ampullary and periampullary lesions. Radiographics 2014;34:624–41. doi: 10.1148/rg.343125191 PMID: 24819785. 24819785

[6] Khoury T, Adileh M, Imam A, Azraq Y, Bilitzky-Kopit A, Massarwa M et al. Parameters suggesting spontaneous passage of stones from common bile duct: a retrospective study. Can J Gastroenterol Hepatol 2019;2019:5382708. doi: 10.1155/2019/5382708 PMID: 30941329; PMCID: PMC6420964. 30941329 PMC6420964

[7] Morosin T, De Robles MSB, Putnis S Gallstone ileus: an unusual cause of intestinal obstruction. Cureus. 2020;12:e7284. doi: 10.7759/cureus.7284 PMID: 32206475; PMCID: PMC7077742. 32206475 PMC7077742

[8] Joshi A, Rajpal K, Kakadiya K, Bansal A Role of CT and MRCP in evaluation of biliary tract obstruction. Curr Radiol Rep 2014;2:72 10.1007/s40134-014-0072-x.

[9] McLaughlin CW, Raines M Obstruction of the alimentary tract from gallstones. The American Journal of Surgery 1951;81:P424–30. doi: 10.1016/0002-9610(51)90254-1. 14819495

[10] McGowan DR, Norris JM, Zia K Vomiting gallstones as a presenting feature of small bowel obstruction secondary to inflammatory stricture. BMJ Case Rep 2013;2013:bcr2013008819. doi: 10.1136/bcr-2013-008819 PMID: 23608850; PMCID: PMC3645219. PMC3645219 23608850

